# The modulation effects of repeated transcutaneous auricular vagus nerve stimulation on the functional connectivity of key brainstem regions along the vagus nerve pathway in migraine patients

**DOI:** 10.3389/fnmol.2023.1160006

**Published:** 2023-06-02

**Authors:** Yiting Huang, Yue Zhang, Sierra Hodges, Hui Li, Zhaoxian Yan, Xian Liu, Xiaoyan Hou, Weicui Chen, Thalia Chai-Zhang, Jian Kong, Bo Liu

**Affiliations:** ^1^Department of Psychiatry, Massachusetts General Hospital, Harvard Medical School, Charlestown, MA, United States; ^2^Department of Radiology, The Second Affiliated Hospital of Guangzhou University of Chinese Medicine, Guangzhou, China; ^3^Department of Neurology, The Second Affiliated Hospital of Guangzhou University of Chinese Medicine, Guangzhou, China

**Keywords:** taVNS, migraine, serotonergic, noradrenergic, NTS

## Abstract

**Background:**

Previous studies have shown a significant response to acute transcutaneous vagus nerve stimulation (taVNS) in regions of the vagus nerve pathway, including the nucleus tractus solitarius (NTS), raphe nucleus (RN) and locus coeruleus (LC) in both healthy human participants and migraine patients. This study aims to investigate the modulation effect of repeated taVNS on these brainstem regions by applying seed-based resting-state functional connectivity (rsFC) analysis.

**Methods:**

70 patients with migraine were recruited and randomized to receive real or sham taVNS treatments for 4 weeks. fMRI data were collected from each participant before and after 4 weeks of treatment. The rsFC analyses were performed using NTS, RN and LC as the seeds.

**Results:**

59 patients (real group: *n* = 33; sham group: *n* = 29) completed two fMRI scan sessions. Compared to sham taVNS, real taVNS was associated with a significant reduction in the number of migraine attack days (*p* = 0.024) and headache pain intensity (*p* = 0.008). The rsFC analysis showed repeated taVNS modulated the functional connectivity between the brain stem regions of the vagus nerve pathway and brain regions associated with the limbic system (bilateral hippocampus), pain processing and modulation (bilateral postcentral gyrus, thalamus, and mPFC), and basal ganglia (putamen/caudate). In addition, the rsFC change between the RN and putamen was significantly associated with the reduction in the number of migraine days.

**Conclusion:**

Our findings suggest that taVNS can significantly modulate the vagus nerve central pathway, which may contribute to the potential treatment effects of taVNS for migraine.

**Clinical Trial Registration**: http://www.chictr.org.cn/hvshowproject.aspx?id=11101, identifier ChiCTR-INR-17010559.

## Introduction

Vagus nerve stimulation has emerged as a potential treatment modality in the management of migraine (Straube et al., 2015; Grazzi et al., 2016; Tao et al., 2018; Zhang et al., 2021). Previous studies have demonstrated a significant response to acute transcutaneous vagus nerve stimulation (taVNS) in regions of the vagus nerve pathway, including the nucleus tractus solitarius (NTS), raphe nucleus (RN) and locus coeruleus (LC) in both healthy human participants and patients with migraine (Frangos et al., 2015; Yakunina et al., 2017, 2018; Sclocco et al., 2019, 2020; Zhang et al., 2019; Borgmann et al., 2021). However, the modulation effect of repeated taVNS on these key brain stem regions has yet to be examined.

Approximately 80% of vagus nerve (VN) fibers are sensory fibers that relay both somatic and general visceral signals (Yuan and Silberstein, 2016). The afferent vagal nerves primarily project to the nucleus tractus solitarius, which in turn transmits the signal to the other brain stem nodes such as the locus coeruleus, raphe nuclei, and other subcortical and cortical regions (Dorr and Debonnel, 2006; Manta et al., 2009, 2012; Sacca et al., 2022).

Literature suggests that the locus coeruleus and raphe nuclei are also associated with two neurotransmitter systems potentially important in regulating migraine (Saper, 2011; Obata, 2017). The first neurotransmitter system is the noradrenergic system, which plays a major role in arousal, attention, and the stress response (Berridge and Spencer, 2017), as well as long-term synaptic plasticity and pain modulation (Benarroch, 2009a,b). The locus coeruleus has the greatest amount of noradrenaline in the central nervous system and provides extremely widespread noradrenergic innervations of the entire cortex, diencephalon, and many other brain structures (Benarroch, 2009a,b). Noradrenergic neurons in the LC and its terminals in the dorsal reticular nucleus (DRt), medial prefrontal cortex (mPFC), spinal dorsal horn, and trigeminal spinal nucleus caudalis (spVc) have been shown to participate in the development and maintenance of allodynia and hyperalgesia after nerve injury (Taylor and Westlund, 2017), in addition to their well-described role in inhibiting spinal nociceptive transmissions. Thus, the LC may be a critical modulator for both pain inhibition and facilitation.

The second neurotransmitter system involved in regulating the pain experience is the serotonergic neuromodulatory system. Literature suggests that serotonin plays a major role in modulating pain perception. For instance, combined serotonin-norepinephrine reuptake inhibitors (SNRI) are increasingly considered to be the most effective treatment in the patients comorbid with depression and migraine (Goadsby et al., 2017; Burch, 2019). In the central nervous system, serotonergic innervation of the cerebral cortex, subcortical structures, and cerebellum mainly originate from the dorsal and median raphe nuclei (RN) (Ren et al., 2018).

The aim of this study is to explore the modulatory effect of repeated taVNS on the resting-state functional connectivity (rsFC) of the NTS, LC, and RN in migraine patients. We hypothesize that these brain stem targets of vagal afferents may be modulated by repeated taVNS.

## Methods

### Participants

70 patients with migraine were recruited and randomized in an equal ratio to receive real or sham taVNS treatments. Migraine was diagnosed by licensed neurobiologists based on the International Classification of Headache Disorders, 2nd Edition (ICHD-II). The study’s protocol was approved by the Institutional Review Board of the Second Affiliated Hospital of Guangzhou University of Chinese Medicine.

The dataset has been previously used to investigate how taVNS stimulation can modulate functional connectivity of subregions of the thalamus (Zhang et al., 2021). This study focuses on how taVNS can modulate the functional connectivity of key regions in the brain stem vagus nerve pathway (NTS, LC, and RN), which has not been published before. Please see supplementary materials or our previous publication (Zhang et al., 2021) for inclusion and exclusion material.

### Experimental procedure

All patients underwent two MRI scans (before and after repeated taVNS). The entire study lasted for about 8 weeks, consisting of a baseline period of 4 weeks collecting migraine attack characteristics prior to treatment and the 4-week treatment period. At enrollment, patients were requested to maintain diary records of their headaches for the duration of the study. Each recorded headache entry included onset time, duration, pain intensity (using Visual Analog Scale (VAS) score), accompanying symptoms, and the use of rescue medication if any. Both real and sham taVNS treatments consisted of 12 sessions in a schedule of 3 sessions per week for 4 weeks, with each session lasting for 30 min. A 1 Hz continuous electrical stimulation was applied at the left cymba concha (Badran et al., 2018) for real taVNS treatment, while the sham stimulation site was located on the left tail of the helix (Badran et al., 2018). See supplementary materials for details in experimental procedures and data acquisition.

### Functional analysis

Functional data preprocessing and statistical analysis were performed in the SPM12-based toolbox CONN19.c (Whitfield-Gabrieli and Nieto-Castanon, 2012),^1^ SUIT (Diedrichsen, 2006),^2^ and AFNI software version 17.2.05 (Cox et al., 2017).^3^ See supplementary materials for data preprocessing procedures.

The bilateral NTS were defined based on a publicly available template provided by the team of Nikos Priovoulos (Priovoulos et al., 2019). The bilateral RN (combination of median and dorsal raphe) and LC seeds were defined based on the Automatic Anatomical Labeling 3 atlas 3 (AAL) template (Pollak Dorocic et al., 2014). In the first-level analysis, we produced a correlation map for each patient by extracting the BOLD time series from each brainstem seed and computing Pearson’s correlation coefficients between the time series in every seed and all other voxels of the whole brain, respectively. Correlation coefficients were Fisher transformed into ‘Z’ scores to increase normality.

Seed-to-voxel second-level analyses were performed using a mixed-designed ANOVA with treatment (real taVNS vs. sham taVNS) entered as the between-subject factor, time (pre-treatment vs. post-treatment) as the within-subject factor, and age and gender as covariates. We also performed a one-sample t-test to assess the baseline functional connectivity of each seed in all migraine patients. A threshold of voxel-wise *p* < 0.005 and cluster-level *p* < 0.05 FDR corrected was applied for second-level analyses.

Based on previous imaging studies, our *a prior* regions of interest included: (1) downstream targets of vagal afferents in the brain stem: LC and RN (Dorr and Debonnel, 2006; Manta et al., 2009, 2012) and (2) the pain perception and modulation network in migraine: hypothalamus, thalamus, hippocampus, amygdala, insula, anterior cingulate cortex (ACC), medial prefrontal cortex (mPFC) and postcentral gyrus (PoCG) (Frangos et al., 2015; Hachem et al., 2018; Kaniusas et al., 2019; Lee et al., 2019; Silberstein et al., 2020; Ashina et al., 2021). An initial threshold of voxel-wise *p* < 0.005 was used in all data analysis. To correct for multiple comparisons, Monte Carlo simulations using the 3dFWHMx and 3dClustSim implemented in AFNI were applied for these ROIs (i.e., brain regions listed above, where for each region, the minimum voxel size required for *p* < 0.05 cluster level *p*-value correction will be indicated as a k value in the results presented below). For the rest of the brain, a voxel-wise threshold of *p* < 0.005 with *p* < 0.05 cluster level FDR correction was applied. A similar analysis was performed in our previous study (Gollub et al., 2018).

To investigate the association between the rsFC change and the primary clinical outcome (number of migraine attack days), we created a sphere with a 2 mm radius using the peak coordinates of the brain regions that produced significant rsFC changes after taVNS and extracted the average *z* values. Then, we performed a correlation analysis between the rsFC z value changes and the corresponding clinical outcomes across all participants.

## Results

### Clinical outcome

59 patients (33 in the taVNS group, 26 in the sham taVNS group) completed two MRI scans before and after 4 weeks of treatment. No patients were excluded due to fMRI head motion correction based on the calculated mean frame displacement (FD) standard.

Compared to the sham taVNS group, patients in the real taVNS treatment group had a significant reduction in the number of migraine attack days ([*F* (1,57) =5.41, *p* = 0.024]), headache pain intensity ([*F* (1,57) = 7.52, *p* = 0.008]), and migraine attack times (numbers) ([*F* (1,57) = 6.29, *p* = 0.015]). There was no significant improvement in MSQ, SAS and SDS evaluation after real taVNS treatment. Detailed patients’ baseline characteristics and clinical outcome measurements were shown in Supplementary Table e1.

### Functional connectivity results

#### NTS functional connectivity results

Among all migraine patients, baseline functional connectivity of the NTS showed positive connectivity with the bilateral angular gyrus (AG), superior/middle frontal gyrus, posterior cingulate gyrus, fusiform gyrus; left hippocampus/parahippocampus, thalamus, cerebellum, and right lingual gyrus; and negative connectivity with the bilateral dorsal lateral prefrontal cortex (dlPFC), putamen, middle/superior temporal gyrus, supramarginal gyrus, supplementary motor cortex, superior frontal gyrus (SFG), left inferior occipital gyrus (IOG), postcentral gyrus (PoCG), and right precentral gyrus (PreCG). Baseline functional connectivity of the NTS is shown in Supplementary Table e2.

**Figure 1:**
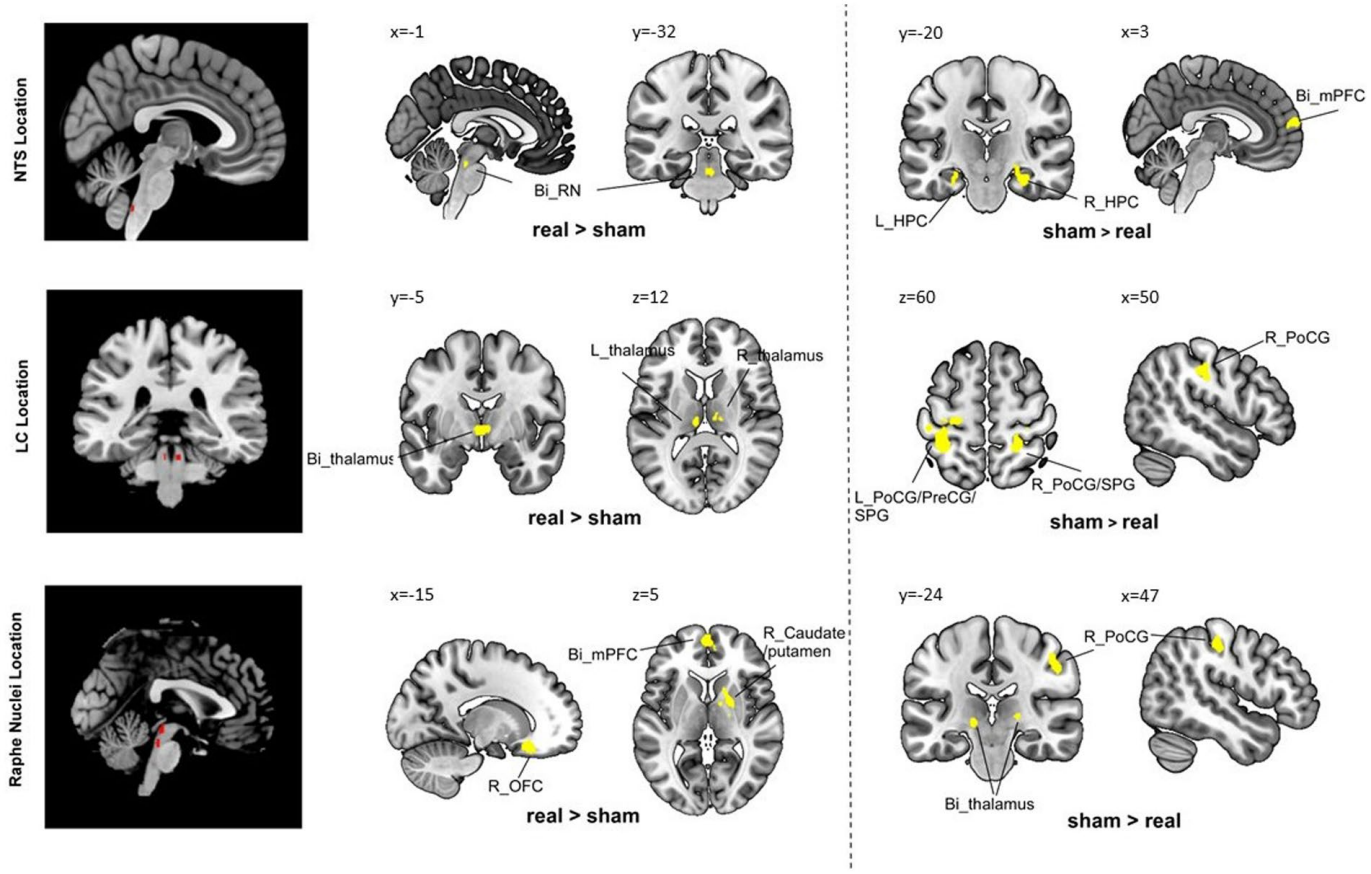
Group comparison results. Bi, bilateral; R, right; L, left; PoCG, postcentral gyrus; PreCG, precentral gyrus; SPG, superior parietal gyrus; AI, anterior insula; OFC, orbital frontal cortex; AG, angular gyrus; mPFC, medial prefrontal cortex; HPC, hippocampus; MCC, middle cingulate cortex.

Compared to the sham group, the real taVNS group showed decreased NTS functional connectivity with the bilateral medial prefrontal cortex (mPFC) and bilateral hippocampus (HPC); and increased functional connectivity with the bilateral raphe nuclei after treatment (Table 1 and Figure 1).

**Table 1:** Seed-based analysis results.

	Condition (Post>pre)	Region	MNI coordinates			Peak *z* value	Cluster size (voxel number)
Seed			X	Y	Z		
NTS	Sham > real	Bi_mPFC	−2	58	10	4.08	155	R_HPC	30	−20	−20	4.65	190	L_HPC	−28	−18	−20	3.29	46	Real > sham	Bi_RN	2	−32	−22	3.27	16
LC	Sham > real	L_SPG	−36	−42	60	4.77	694	L_PoCG/PreCG	−24	−24	52	4.12	R_PoCG	48	−20	38	4.36	131	R_PoCG/SPG	24	−42	62	3.86	234	Real > sham	Bi_Thalamus	−4	−6	0	4.74	115	R_Thalamus	16	−16	6	3.71	56	L_Thalamus	−8	−22	10	3.48	34
RN	Sham > real	R_PoCG	48	−24	46	3.59	82	R_Thalamus	22	−24	2	3.23	32	L_Thalamus	−16	−24	−2	3.64	22	Real > sham	L_OFC	−14	22	−16	4.24	164	L_AG	−52	−58	46	3.77	188	Bi_mPFC/ACC	0	58	4	3.74	122	R_caudate	18	12	12	3.36	219	R_putamen	20	14	−2	2.70

Bi, bilateral; R, right; L, left; NTS, nucleus tractus solitarius; LC, locus coeruleus; RN, Raphe Nuclei; PoCG, postcentral gyrus; PreCG, precentral gyrus; SPG, superior parietal gyrus; AI, anterior insula; OFC, orbital frontal cortex; AG, angular gyrus; mPFC, medial prefrontal cortex; ACC, anterior cingulate cortex; HPC, hippocampus; MCC, middle cingulate cortex.

#### LC functional connectivity results

Baseline functional connectivity of the LC showed positive connectivity with the bilateral cerebellum, lingual gyrus, and hippocampus/parahippocampus, and negative connectivity with right occipital fusiform gyrus (Supplementary Table e2).

Compared to the sham group, the real taVNS group showed decreased LC functional connectivity with the bilateral postcentral gyrus (PoCG), superior parietal gyrus (SPG), and left precentral gyrus (PreCG); and increased functional connectivity with the bilateral thalamus after treatment (Table 1 and Figure 1).

#### RN functional connectivity results

Baseline functional connectivity of the RN showed positive connectivity with the left thalamus and negative connectivity with the left caudate and right occipital pole (Supplementary Table e2).

Compared to the sham group, the real taVNS group showed decreased RN functional connectivity with the bilateral thalamus and right PoCG; and increased functional connectivity with the bilateral mPFC/anterior cingulate cortex (ACC), left occipital frontal cortex (OFC), left angular gyrus (AG), right caudate, and putamen after treatment (Table 1 and Figure 1).

#### Association with primary clinical outcome

**Figure 2:**
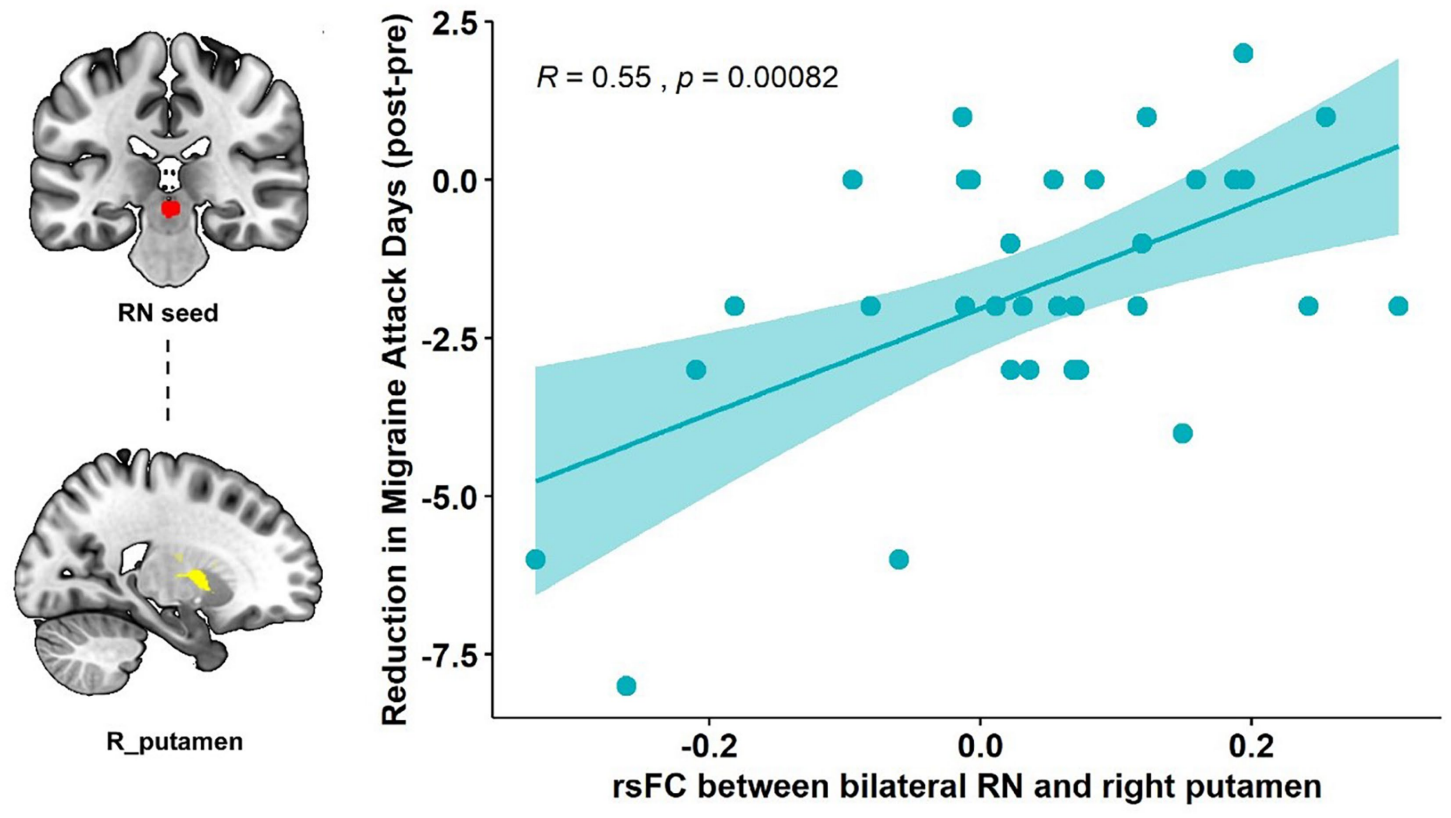
Correlation results of the RN-putamen rsFC change and clinical outcomes. The scatterplot indicates the significant correlation between the change in number of migraine attack days and the rsFC change of the bilateral RN and right putamen after real taVNS treatment (*R* = 0.55, *p* < 0.001, significant after Bonferroni correction).

Pearson’s correlation showed that the RN – right putamen rsFC change was significantly correlated with the primary outcome (reduction of the number of migraine days, *R* = 0.55, *p* < 0.001, *p* = 0.015 after Bonferroni correction) in the real taVNS group after treatment (Figure 2).

There is no significant association between NTS/LC rsFC change and the corresponding primary clinical outcome changes.

## Discussion

In the present study, we investigated the modulatory effects of repeated taVNS on functional connectivity of the NTS, LC and RN in patients with migraine. Comparisons of clinical measurements indicated repeated taVNS could significantly relieve patients’ symptoms. Functional analysis showed that repeated taVNS could modulate the connection between the NTS and key brain regions in the limbic system (bilateral hippocampus), and the functional connectivity of both the LC and RN with brain regions closely relating to pain processing and modulation (bilateral postcentral gyrus/bilateral thalamus/mPFC). Our findings also demonstrated that repeated taVNS could strengthen the functional connectivity between the RN and caudate/putamen, the main dopaminergic projecting targets. The RN-caudate/putamen rsFC change was significantly associated with the reduction in the number of migraine attack days. These findings suggests that repeated taVNS could significantly modulate the vagus nerve pathway in patients with migraine.

As the major target of vagal afferents, the NTS receives direct inputs from the vagus nerve and subsequently projects to other brainstem regions including the LC, RN and parabrachial nucleus, along with the forebrain limbic structures such as the periventricular nucleus of the hypothalamus, the thalamus, the central nucleus of the amygdala, the hippocampus, the bed nucleus of the stria terminalis, and the nucleus accumbens (Zoccal et al., 2014). In the current study, we observed a significantly decreased NTS rsFC with the bilateral mPFC and hippocampus and increased rsFC with the RN after the real taVNS treatments compared to the sham.

The hippocampus is a main structure of the limbic system. Accumulating evidence suggests that limbic system, and the hippocampus alone, is involved in pain processing, pain-related attention, anxiety, and the stress response (Liu and Chen, 2009; Mokhtari et al., 2019; Yu et al., 2020). Both structural and functional abnormalities of the hippocampus have been reported in migraine patients (Biggio et al., 2009). Literature suggests that headache frequency, cumulative number of migraine attacks, anxiety scores, depression scores, and genetic variants are related to hippocampal morphology and functional changes in individuals with migraine (Liu et al., 2018). A previous neuroimaging study found that migraineurs had greater pain-induced activation of the hippocampus, and the activation strength had a significant correlation with headache frequency in migraine patients (Schwedt et al., 2014). Our results suggest that taVNS could modulate the functional connection between the hippocampus and the NTS. This finding is also consistent with a previous study in which the authors found that plasticity within the hippocampus is an important contributor to the vagus nerve response (Biggio et al., 2009).

We found that taVNS could modulate the rsFC of both the LC and RN with brain regions closely relating to pain processing and modulation (PoCG/thalamus/mPFC). In particular, both the LC and RN is associated with a reduced functional connectivity with the PoCG after repeated taVNS treatment. Previous studies have identified involvement of the PoCG in chronic pain (Kong et al., 2013; Ossipov et al., 2014). Particularly, the majority of patients with migraine show somatosensory hypersensitivity (i.e., reduced cutaneous pain thresholds) and other symptoms of somatosensory hypersensitivity (i.e., cutaneous allodynia) during migraine attacks (Schwedt, 2013). The LC sends strong projections to the sensory cortex and has long been posited to play an important role in modulating sensory encoding (Bruinstroop et al., 2012; Vazey et al., 2018).

As the major source of serotonergic projections, the raphe nucleus plays a role in the regulation of multiple functional systems, including the somatosensory system (Hornung, 2003). Our results suggest that repeated taVNS may regulate the perception of nociceptive inputs in patients with migraine by interfering with the connection between LC/RN and PoCG. These connectivity changes may alter the projection of noradrenergic and serotonergic neurotransmitters in the brain stem to the somatosensory cortex.

The thalamus is a major center in the brain for processing nociceptive information and relays this information to cortical regions for further processing (Lenz et al., 2004). Evidence of activation of the thalamus during migraines is clear and well established (Bahra et al., 2001; Afridi et al., 2005; Tu et al., 2019). It is worth noting that our results showed that repeated taVNS could modulate the rsFC of both the LC and RN with the thalamus in patients with migraine. A previous study found that both noradrenergic and serotonergic fibers innervate the thalamus, and the origins of these projections are primarily located at the LC and RN (Westlund et al., 1991). We also found that taVNS can significantly modulate the connectivity between the thalamus and sensory-motor cortex (Zhang et al., 2021). Interestingly, we found that after repeated taVNS, the rsFC of the thalamus was increased with the LC but decreased with the RN. As the noradrenergic and serotonergic fibers project to multiple nuclei of the thalamus, involving both excitatory and inhibitory effects (Varela, 2014), future studies are needed to investigate more details on how taVNS may affect the LC and RN projections to the thalamus.

We also found that taVNS could significantly modulate the rsFC between the RN and bilateral mPFC / ACC in patients with migraine. The mPFC is a crucial brain region that integrates information from multiple cortical and subcortical areas and converges updated outputs to downstream structures. In addition, the mPFC / ACC is also a key region of the descending pain modulation system (Kong et al., 2010). The functional and structural alterations of the mPFC in patients with migraine have been reported (Jin et al., 2013; Soheili-Nezhad et al., 2019). It was considered to play an essential role in regulating pain-related emotion (depression and anxiety) and cognition in patients with migraine (Ma et al., 2018; Tu et al., 2019; Zhang et al., 2019). A previous study showed that migraine is associated with increased PAG – mPFC connectivity, and the increased connectivity decreased after acupuncture treatments (Li et al., 2016). We also found taVNS can modulate the PAG – ACC/mPFC connectivity in patient with migraine (Cao et al., 2021). Interestingly, we explored the association between the RN-mPFC rsFC change and changes in depression scores in the repeated taVNS treatment group. The results showed that these two factors are highly correlated, with a *p* value of 0.0038 (*R* = −0.49). Our result suggests that taVNS may hold the potential to relieve both headache and depression in patients comorbid with migraine and depression. This is consistent with our previous studies showing that taVNS can also reduce symptoms in patients with depression (Kong et al., 2018; Li et al., 2022).

Our results showed that repeated taVNS significantly altered the functional connectivity between the RN and basal ganglia subregions, specifically the right putamen and caudate. This connectivity change is associated with a reduction in the number of migraine attack days, i.e., the higher the functional strength between RN and right putamen, the greater the reduction in the number of attack days in migraine patients.

Literature has suggested that the basal ganglia may be involved in the sensory, emotional / cognitive, and endogenous/modulatory domains of pain processing (Borsook et al., 2010). In particular, the putamen is one of the major sites of cortical input into basal ganglia loops and is frequently activated during pain (Starr et al., 2011). Dysfunctions of the monoamine systems, especially the dopaminergic and serotonergic projections to the basal ganglia, were reported to correlate with individual ratings of the sensory and unpleasant qualities of the experience of pain(Scott et al., 2006) and might be the early result of chronic maladaptation to persistent pain (Sagheddu et al., 2015). Thus, we speculate that the repeated administration of taVNS might modulate pain perception attributed to the serotonin activation in the putamen projected from the RN in patients with migraine.

There are several limitations to this study. Firstly, the current study was conducted over a 4-week period and therefore the effects observed can only represent short-and mid-term effects; a study with a longer duration is needed to investigate long-term effects. Secondly, the sham stimulation was applied at the tail of the helix with the same electrical stimulation parameters, which might also activate the sensory related cortex. Caution needs to be taken in the interpretation of the comparison results of the rsFC change between the two groups, especially when it comes to the upregulation of somatosensory cortex. Future studies are needed to validate / replicate our findings.

In conclusion, we found that repeated taVNS can modulate the connection between the key nodes of the brain stem vagus nerve pathway with the limbic system and pain perception and modulation networks. These findings suggest that repeated taVNS may yield pain relief through modulating of the NTS, LC, and RN connections with the above regions, which may further affect their synaptic plasticity and neurotransmitter release associated with migraine.

## Data availability statement

The raw data supporting the conclusions of this article will be made available per request.

## Ethics statement

The studies involving human participants were reviewed and approved by the Institutional Review Board of the Second Affiliated Hospital of Guangzhou University of Chinese Medicine. The patients/participants provided their written informed consent to participate in this study.

## Author contributions

YZ, BL, and JK: design and conceptualized study. YH and JK: analyze and interpret the data. YZ, HL, ZY, XL, XH, and WC: acquisition of data. YH, SH, TC-Z, and JK: manuscript preparation. YZ and BL: funding acquisition. All authors contributed to the article and approved the submitted version.

## Funding

This study was supported by the Medical Scientific Research Foundation of Guangdong Province of China (A2017234) and the Administration of Traditional Chinese Medicine of Guangdong Province of China (20182047).

## Conflict of interest

The authors declare that the research was conducted in the absence of any commercial or financial relationships that could be construed as a potential conflict of interest.

## Publisher’s note

All claims expressed in this article are solely those of the authors and do not necessarily represent those of their affiliated organizations, or those of the publisher, the editors and the reviewers. Any product that may be evaluated in this article, or claim that may be made by its manufacturer, is not guaranteed or endorsed by the publisher.

## Footnotes

^1^https://web.conn-toolbox.org/

^2^https://www.diedrichsenlab.org/imaging/suit.htm

^3^https://afni.nimh.nih.gov
