# Supplementary material for: The modulation effects of repeated transcutaneous auricular vagus nerve stimulation on the functional connectivity of key brainstem regions along the vagus nerve pathway in migraine patients
Source: Front Mol Neurosci. 2023 Jun 2;16:1160006. doi: 10.3389/fnmol.2023.1160006 (PMC10275573; doi:10.3389/fnmol.2023.1160006)
Supplement: Supplementary file 1 [file Data_Sheet_1.pdf]

Supplementary material for:

The modulation effects of repeated transcutaneous auricular vagus nerve stimulation on the functional connectivity of key brainstem regions along the vagus nerve pathway in migraine patients

Yiting Huang 1, †; Yue Zhang 2, †; Sierra Hodges 1 ; Hui Li 3; Zhaoxian Yan 2; Xian Liu 2; Xiaoyan Hou 2; Weicui Chen 2; Thalia Chai-Zhang1; Jian Kong1; Bo Liu 2

1Department of Psychiatry, Massachusetts General Hospital, Harvard Medical School, 120 2nd Ave, Room 101, Charlestown, MA 02129, USA

2Department of Radiology, The Second Affiliated Hospital of Guangzhou University of Chinese Medicine, Guangzhou 510120, China

3 Department of Neurology, The Second Affiliated Hospital of Guangzhou University of Chinese Medicine, Guangzhou 510120, China

† These authors contributed equally to the manuscript.

Participants

All patients were recruited from the Second Affiliated Hospital of Guangzhou University of Chinese Medicine between May 2017 to May 2019. The study's protocol was approved by the Institutional Review Board of the Second Affiliated Hospital of Guangzhou University of Chinese Medicine and registered in the Chinese Clinical Trial Registry (ChiCTR-INR-17010559, February 7, 2017, http://www.chictr.org.cn/hvshowproject.aspx?id=11101). Informed consent was obtained from all participants.

Migraine was diagnosed by licensed neurobiologists based on the International Classification of Headache Disorders, 2nd Edition (ICHD-II). Inclusion criteria was: 1. Between 18-45 years of age; 2. Right-handedness; 3. At least six months of migraines reported; 4. At least two headache attacks on average per month; 5. No usage of prophylactic headache medications in the past month; 6. No usage of psychoactive or vasoactive drugs for at least three months prior to enrollment.

Exclusion criteria was defined as: 1. Headaches attributable to other diseases; 2. Pregnancy or lactation; 3. Other chronic pain conditions; 4. Severe head deformities or intracranial lesions; 5. Score of >50 on the Self-Rating Anxiety Scale (SAS) or Self-Rating Depression Scale (SDS).

Experimental paradigms

70 migraine patients were recruited and randomized in an equal ratio to receive real taVNS or sham taVNS treatment from the outpatient unit of the Department of Neurology in the Second Affiliated Hospital of Guangzhou University of Chinese Medicine with prescreening by neurologists. All participants were blinded to the treatment they received (real vs sham).

Patients were studied for 8 weeks, consisting of a baseline period of 4 weeks prior to treatment and the 4-week treatment period. At enrollment, patients were requested to maintain full diary records of their headaches for the duration of the study. Each recorded headache entry included onset time, duration, pain intensity (using Visual Analog Scale (VAS) score), accompanying symptoms, and the use of rescue medication if any.

Stimulation procedure

Stimulation was applied by trained physicians using electronic acupuncture treatment instruments (Hwato SDZII, Suzhou Medical Supplies Factory, Suzhou, China) at the outpatient clinic of the Department of Neurology in the Second Affiliated Hospital of Guangzhou University of Chinese Medicine. A continuous wave (frequency: 1 Hz; width: ~0.2 ms) was applied. Stimulation intensity was calibrated to be the strongest sensation without pain (approximately 1.5–5 mA). Both real and sham taVNS treatments consisted of 12 sessions in a schedule of 3 sessions per week for 4 weeks, with each session lasting for 30 minutes.
Similar to our previous studies [1], the real stimulation site for taVNS was located at the left cymba concha [2], while the sham stimulation site was located on the left tail of the helix. These stimulation sites were selected based

on findings from a previous anatomical dissection study which reported that the cymba concha is innervated by the auricular branch of the vagus nerve (ABVN) in 100% of the exposed auricle, contrasted to the tail of the helix which lacks any cutaneous vagal innervation [3].

Clinical outcomes and statistical analysis

All patients completed the self-recorded headache diaries for the baseline period (weeks 1 to 4) and the treatment period (weeks 5 to 8). We selected mean reduction in the number of migraine days as our primary outcome based on a previous study using taVNS as a treatment for migraine [4].

Secondary outcomes included: (i) mean reduction in pain intensity of each migraine measured by the 0-10 visual analog scale (VAS); (ii) mean reduction in duration of migraine attacks; (iii) increase in score on the Migraine Specific Quality-of-Life Questionnaire (MSQ); (iv) reduction on the Zung Self-rating Depression Scales (SDS) and/or Zung Self-rating Anxiety Scales (SAS). All staff tasked with collecting clinical measurements were blinded to the subject treatment distribution.

Estimation of taVNS effectiveness was conducted by comparing changes in migraine days using a mixed model regression with time, group allocation, and interaction between the two as fixed effects and the patient as a random effect. Age and gender were included as covariates. The analysis was performed using R Version 3.1.0, with lme4 [5] and lmerTest [6]. A similar analysis was performed on the secondary clinical outcomes, including pain intensity, frequency of migraine attack times, MSQ, SDS, and SAS.

Functional magnetic resonance imaging data acquisition

All patients participated in identical functional magnetic resonance imaging (fMRI) scanning sessions before and after 4 weeks of treatment. All magnetic resonance imaging/functional magnetic resonance imaging (MRI/fMRI) scans were conducted on a 3.0T Siemens MRI scanner (Siemens MAGNETOM Verio 3.0 T, Erlangen, Germany) with a 24-channel phased-array head coil.

Resting state functional MRI encompassing the whole brain was acquired with the following parameters: TR = 2000 ms, TE = 30 ms, FOV = 224 mm× 224 mm, matrix = 64 × 64, flip angle = 90°, slice thickness = 3.5 mm, interslice gap = 0.7 mm, 31 axial slices paralleled, and 240 time points. Subjects were instructed to stay awake, remain motionless, and keep their eyes closed during the 8-minute resting-state fMRI scan. T1-weighted high-resolution structural images were applied with the following parameters: TR = 1900 ms, TE = 2.27 ms, flip angle = 9°, FOV = 256 mm× 256 mm, matrix = 256× 256, and slice thickness = 1.0 mm.

Functional analysis

Whole-brain preprocessing

Whole-brain functional imaging data were preprocessed using the MATLAB/SPM-based toolbox CONN19.c[7]. The whole-brain preprocessing steps included realignment, slice timing correction, segmentation of structural data, spatial and functional normalization into standard stereotactic MNI space, reslicing into 2 × 2 × 2 mm voxels and smoothed at a Gaussian kernel of 6mm Full Width at Half Maximum (FWMH) for seed-based correlation analysis (SCA).

Brainstem preprocessing

For brainstem preprocessing, we used the SUIT toolbox[8] to improve normalization and more precisely define regions of interests (ROIs) for the subsequent time series extraction. Specifically, structural images were first reoriented to LPI-orientation and centered at (0,0,0). Functional images were realigned, slice timing corrected, centered at (0,0,0), and then co-registered to the structural images. Using the SUIT toolbox, the following procedures were applied: i) segmentation of the whole-brain images as implemented in SPM12, ii) cropping of the images, retaining only the brainstem and cerebellum, iii) normalization using the DARTEL (diffeomorphic anatomical registration through exponentiated lie algebra) engine [9] that uses gray and white-matter segmentation maps produced during cerebellar isolation to generate a flow field using Large Deformation Diffeomorphic Metric Mapping [10], and (iv) reslicing to a voxel size of 2 × 2 × 2 mm^3. Similar to a previous study [11], we did not smooth the normalized images due to the small size of the brainstem nuclei and their close anatomical locations.

In order to minimize the effects of head motion, subjects with a mean frame displacement (FD) in excess of 0.2 mm were excluded. [12,13] (No subjects were excluded in the data analysis). Outlier time points were identified in the motion parameters and global signal intensity using the ART implementation in CONN toolbox (https://www.nitrc.org/projects/artifact_detect/). Confounding factors were removed using linear regression utilizing WM & CSF signals, linear trend, subject motion (6 rotation/translation motion parameters and 6 first-order temporal derivatives), and outliers (scrubbing). Then the residual BOLD time series was band-pass filtered with a frequency window of 0.008-0.09 Hz.

Concerning the relatively deep location of the brainstem seeds, we also calculated the temporal signal-to-noise ratio (tSNR) of three seeds using the Matlab/SPM12 based toolbox MRIqual. There is no significant difference of the tSNR found in NTS(p=0.462), LC(p=0.928), and RN(p=0.839) at baseline. In addition, the two-way mixed ANOVA was performed to evaluate the effects of group and condition on the tSNR of the seeds. There is no statistically significant two-way interactions and main effects of group and condition for tSNR of the seed NTS, LC and RN. Please see Table e3 and Table e4 below for detailed results.

Table e1 Clinical outcome measurements in the real and sham taVNS groups.

	Sham Group, n=26			taVNS Group, n=33			Interaction effect
	Pre-treatment mean (sd)	Post-treatment mean (sd)	Post-pre [95% CI]	Pre-treatment mean (sd)	Post-treatment mean (sd)	Post-pre [95% CI]	
Migraine days	4.0 (3.2)	3.2 (2.3)	-0.7 [-2.1; 0.6]	4.0 (1.9)	1.5 (1.4)	-2.5 [-3.3; -1.6]	F(1,57)=5.41, p=0.024
Pain intensity	51.6 (15.2)	47.6 (18.2)	-4.1 [-9.4; 1.3]	50.2 (14.5)	32.8 (20.7)	-17.4 [-25.2; -9.7]	F(1,57)=7.52, p=0.008
Attack times	3.8 (2.4)	4.3 (3.4)	0.4 [-0.9; 1.7]	4.0 (2.3)	2.5 (2.3)	-1.5 [-2.3; -0.6]	F(1,57)=6.29, p=0.015
MSQ	55.9 (10.9)	67.3 (11.6)	11.4 [7.0; 15.8]	57.1 (9.7)	70.8 (10.6)	13.6 [9.1; 18.2]	F(1,57)=0.51, p=0.479
SAS	43.2 (5.0)	40.3 (7.4)	-2.7 [-4.7; -0.7]	43.3 (6.2)	40.2 (7.1)	-3.0 [-4.5; -1.6]	F(1,57)=0.08, p=0.773
SDS	44.6 (5.1)	43.5 (8.6)	-1.0 [-4.0; 2.1]	43.9 (6.2)	41 (6.1)	-2.9 [-4.5; -1.4]	F(1,57)=1.57, p=0.215

Notes: Abbreviations: MSQ = migraine-specific quality of life questionnaire; SAS = Zung self-rating anxiety scale; SDS = Zung self-rating anxiety scale; CI = confidence interval

Table e2 functional connectivity of three brain stem regions at baseline

Seed	Condition	Subject effects	Region	MNI coordinates X	Y	Z	Peak z value	Cluster size
NTS	pre	All subjects (+)	L_MFG	-32	28	42	3.22	148
			L_AG	-46	-52	40	3.98	124
			Bi_mPFC	-2	48	36	3.78	147
		All subjects (-)	None					
		Sham > real	None					
		Real > sham	None					
	post > pre	Sham (+)	None					
		Sham (-)	None					
	post > pre	Real (+)	None					
		Real (-)	None					
LC	pre	All subjects (+)	None					
		All subjects (-)	None					
		Sham > real	None					
		Real > sham	L_ITG	-48	-64	-4	4.75	162
			L_SPG	-32	-44	52	4.57	452
			R_SPG	24	-40	62	4.43	312
			R_PoCG	56	-12	22	4.33	268
	post > pre	Sham (+)	R_calcarine/SOG	24	-80	22	3.98	253
			L_IOG	-46	-70	8	3.91	241
			L_AG	-42	-60	38	3.73	169
			R_PoCG	54	-8	26	3.7	183
		Sham (-)	Bi_precuneus	-8	-42	52	4.28	424
	post > pre	Real (+)	Bi_cuneus/SOG	24	-78	22	3.94	198
		Real (-)	Bi_precuneus	-10	-38	48	3.96	196
RN	pre	All subjects (+)	L_SPG	-24	58	20	4.11	131
		All subjects (-)	None					
		Sham > real	L_OFC	-18	24	-20	4.42	111
			L_IOG	-36	-88	2	3.87	133
		Real > sham	None					
	post > pre	Sham (+)	None					
		Sham (-)	R_putamen/pallidum	24	2	2	4.38	117
			L_STG	-62	-18	-4	4.09	102
			L_putamen/pallidum	-26	-4	2	3.9	164
	post > pre	Real (+)	None					
		Real (-)	None					

MFG: middle frontal gyrus; AG; angular gyrus; Mpfc: medial prefrontal cortex; SOG: superior occipital gyrus; IOG: inferior occipital gyrus; SPG: superior parietal gyrus; STG: superior temporal gyrus; OFC: orbital frontal cortex

Table e3. calculation results of temporal signal-to-noise ratio(tSNR) of the seeds

Group	Condition	NTS	LC	RN
Sham (n=26)	Before	64.5±18.3	93.6±25.9	99.4±30.3
	After	59.0±17.1	94.5±31.5	93.5±32.7
Real(n=33)	Before	60.5±22.7	92.9±38.6	97.7±31.6
	After	65.5±19.1	93.0±31.7	94.3±31.9

Table e4. the tSNR results of two-way mixed measure ANOVA

Effect	NTS	LC	RN
Group	F(1, 57) = 0.084, p =0.773	F(1, 57) = 0.026, p =0.872	F(1, 57) = 0.003, p =0.955
Condition	F(1, 57) = 0.011, p =0.918	F(1, 57) = 0.010, p =0.920	F(1, 57) = 1.161, p =0.286
Group: condition	F(1, 57) = 3.921, p =0.053	F(1, 57) = 0.004, p =0.947	F(1, 57) = 0.085, p =0.772

Reference

1. Zhang Y, Liu J, Li H, Yan Z, Liu X, Cao J, Park J, Wilson G, Liu B, Kong J (2019) Transcutaneous auricular vagus nerve stimulation at 1 Hz modulates locus coeruleus activity and resting state functional connectivity in patients with migraine: An fMRI study. NeuroImage: Clinical 24:101971. doi:https://doi.org/10.1016/j.nicl.2019.101971
2. Badran BW, Brown JC, Dowdle LT, Mithoefer OJ, LaBate NT, Coatsworth J, DeVries WH, Austelle CW, McTeague LM, Yu A, Bikson M, Jenkins DD, George MS (2018) Tragus or cymba conchae? Investigating the anatomical foundation of transcutaneous auricular vagus nerve stimulation (taVNS). Brain Stimul 11 (4):947-948. doi:10.1016/j.brs.2018.06.003
3. Peuker ET, Filler TJ (2002) The nerve supply of the human auricle. Clinical anatomy (New York, NY) 15 (1):35-37. doi:10.1002/ca.1089
4. Straube A, Ellrich J, Eren O, Blum B, Ruscheweyh R (2015) Treatment of chronic migraine with transcutaneous stimulation of the auricular branch of the vagal nerve (auricular t-VNS): a randomized, monocentric clinical trial. The journal of headache and pain 16:543. doi:10.1186/s10194-015-0543-3
5. Bates D, Mächler M, Bolker B, Walker S (2015) Fitting Linear Mixed-Effects Models Using lme4. 2015 67 (1):48. doi:10.18637/jss.v067.i01
6. Kuznetsova A, Brockhoff PB, Christensen RHB (2017) lmerTest Package: Tests in Linear Mixed Effects Models. 2017 82 (13):26. doi:10.18637/jss.v082.i13
7. Whitfield-Gabrieli S, Nieto-Castanon A (2012) Conn: A Functional Connectivity Toolbox for Correlated and Anticorrelated Brain Networks. Brain connectivity 2 (3):125-141. doi:10.1089/brain.2012.0073
8. Diedrichsen J (2006) A spatially unbiased atlas template of the human cerebellum. Neuroimage 33 (1):127-138. doi:10.1016/j.neuroimage.2006.05.056
9. Ashburner J (2007) A fast diffeomorphic image registration algorithm. NeuroImage 38 (1):95-113. doi:10.1016/j.neuroimage.2007.07.007

10. Beg MF, Miller MI, Trouvé A, Younes L (2005) Computing Large Deformation Metric Mappings via Geodesic Flows of Diffeomorphisms. International Journal of Computer Vision 61 (2):139-157. doi:10.1023/B:VISI.0000043755.93987.aa
11. Bär KJ, de la Cruz F, Schumann A, Koehler S, Sauer H, Critchley H, Wagner G (2016) Functional connectivity and network analysis of midbrain and brainstem nuclei. NeuroImage 134:53-63. doi:10.1016/j.neuroimage.2016.03.071
12. Jenkinson M, Bannister P, Brady M, Smith S (2002) Improved optimization for the robust and accurate linear registration and motion correction of brain images. Neuroimage 17 (2):825-841
13. Yan CG, Cheung B, Kelly C, Colcombe S, Craddock RC, Di Martino A, Li Q, Zuo XN, Castellanos FX, Milham MP (2013) A comprehensive assessment of regional variation in the impact of head micromovements on functional connectomics. Neuroimage 76:183-201. doi:10.1016/j.neuroimage.2013.03.004
